# Supplementary material for: EVR: reconstruction of bacterial chromosome 3D structure models using error-vector resultant algorithm
Source: BMC Genomics. 2019 Oct 15;20:738. doi: 10.1186/s12864-019-6096-0 (PMC6794827; doi:10.1186/s12864-019-6096-0)
Supplement: Supplementary file 1 — Additional file 1. Supplementary materials for the evaluation of EVR algorithm. [file 12864_2019_6096_MOESM1_ESM.pdf]

EVR: Reconstruction of Bacterial Chromosome 3D Structure Models Using Error-Vector Resultant Algorithm

Kang-Jian Hua and Bin-Guang Ma*

Hubei Key Laboratory of Agricultural Bioinformatics, College of Informatics, State Key Laboratory of Agricultural Microbiology, Huazhong Agricultural University, Wuhan 430070, China

* Correspondence: mbg@mail.hzau.edu.cn

Supplementary Information

Algorithm evaluation with standard toroidal spiral structures

For algorithm evaluation, standard toroidal spiral structures were generated with different numbers of vertices. The generation equation is as follows:

$$X_i = \left(a \cdot \sin\left(c \cdot \frac{i}{N}\right) + b\right) \cdot \cos\left(\frac{i}{N}\right),$$

$$Y_i = \left(a \cdot \sin\left(c \cdot \frac{i}{N}\right) + b\right) \cdot \sin\left(\frac{i}{N}\right),$$

$$Z_i = a \cdot \cos\left(c \cdot \frac{i}{N}\right),$$

$$i = 1 \dots N,$$

where X_i, Y_i and Z_i represent the 3D coordinates of vertex i, N is the total number of vertexes, and parameters a, b, c represent the radius of the small ring, the radius of the big ring, the number of spirals, respectively. An example of toroidal spiral structure with 500 vertices (parameter setting: $a = 0.5$, $b = 1$ and $c = 20$) is shown in **Figure S1**.

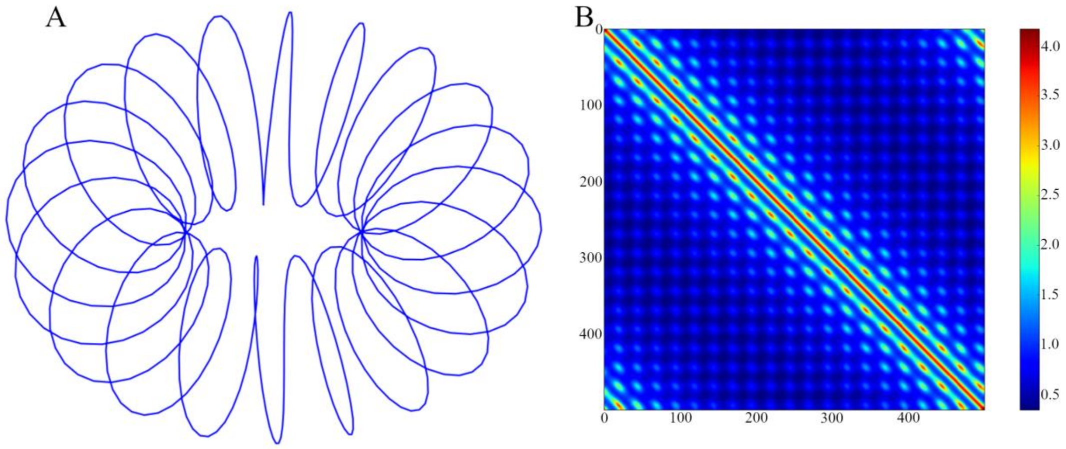

Figure S1. An example of standard structure for algorithm evaluation. (A) A toroidal spiral structure of 500 vertices. (B) The hot map of the simulated interaction frequency (IF) matrix of this structure. Warmer color indicates higher interaction (smaller distance).

With the simulated IF matrix, the toroidal spiral structure can be reconstructed by using the four software tools EVR, miniMDS, ShRec3D and MOGEN. The reconstructed structures were scaled, aligned and superposed with the original standard structure for the calculation of the smallest RMSD value for each software tool (**Figure S2**). As shown in **Figure S2**, our EVR algorithm shows the smallest RMSD value among the four software tools, indicating that our EVR algorithm is accurate.

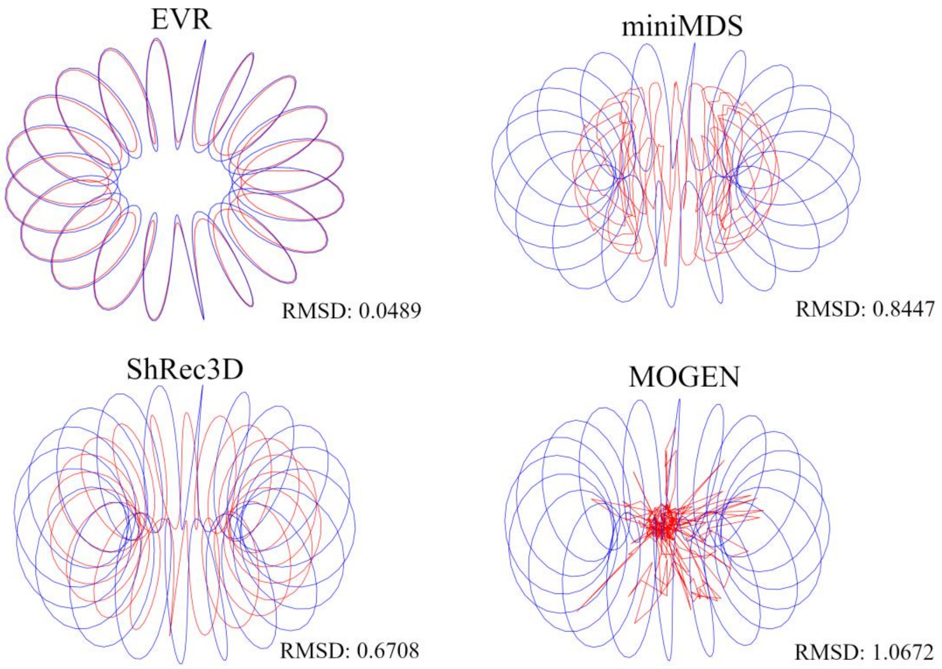

Figure S2. Comparison between the reconstructed structures (red) and the original structure (blue). As can be seen, EVR has the smallest RMSD value compared with miniMDS, ShRec3D and MOGEN, showing the accuracy of our EVR algorithm.

For testing the robustness of reconstruction, difference levels of noises are added to the IF matrix of the standard toroidal spiral structure by randomly selecting values from a range of $-0.5 \times P \times IF_{max}$ to $0.5 \times P \times IF_{max}$, where P is noise level and IF_{max} is the maximum value in IF matrix. An example of reconstructed structure by EVR based on an IF matrix with70% noise level was shown in **Figure S3**.

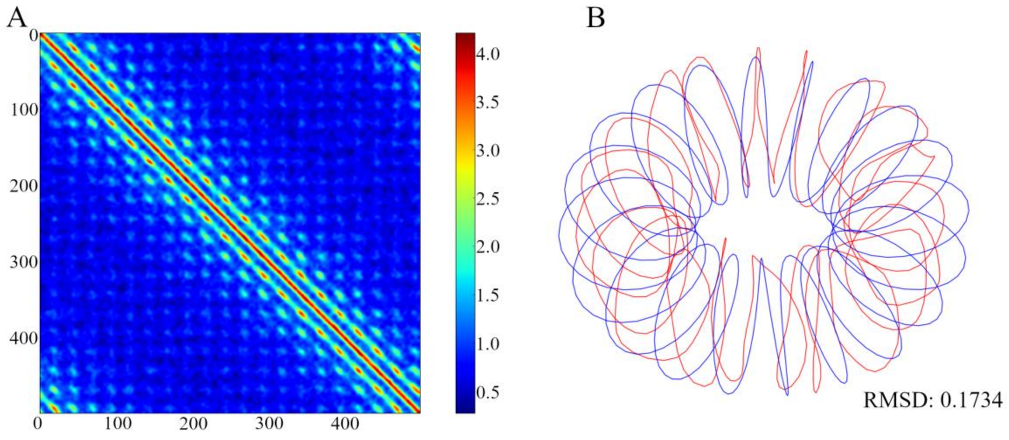

Figure S3. (A) The simulated IF matrix of a toroidal spiral structure with 70% noise level. (B) Comparison between the reconstructed structure (red) by EVR and the original structure (blue). As can be seen, the reconstructed structure is quite similar to the original structure (RMSD: 0.1734), indicating that our EVR algorithm is robust.

See **Figure 4** in the main text for the comparison of robustness among different software tools. If the noise level becomes even larger, for example, noise level = 200%, the RMSD values of all the four software tools become large including our EVR (**Figure S4**). Nonetheless, it seems that EVR still has the smallest RMSD values at such a level of noise averagely.

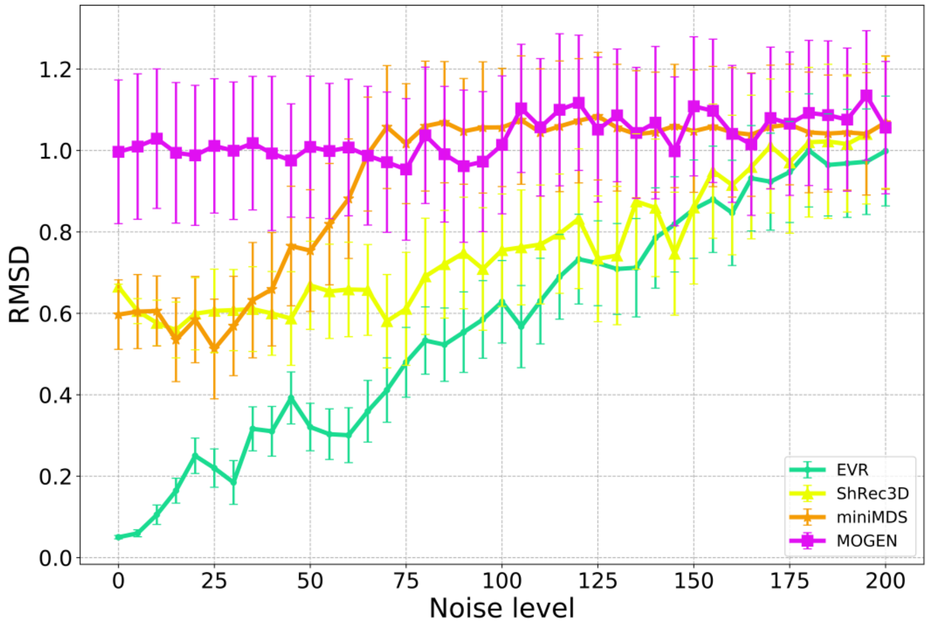

Figure S4. Comparison between reconstructed structures (from noisy data) and original structures using four software tools (up to a noise level of 200%). Both the absolute RMSD value and its trend with the increase of noise level should be considered.

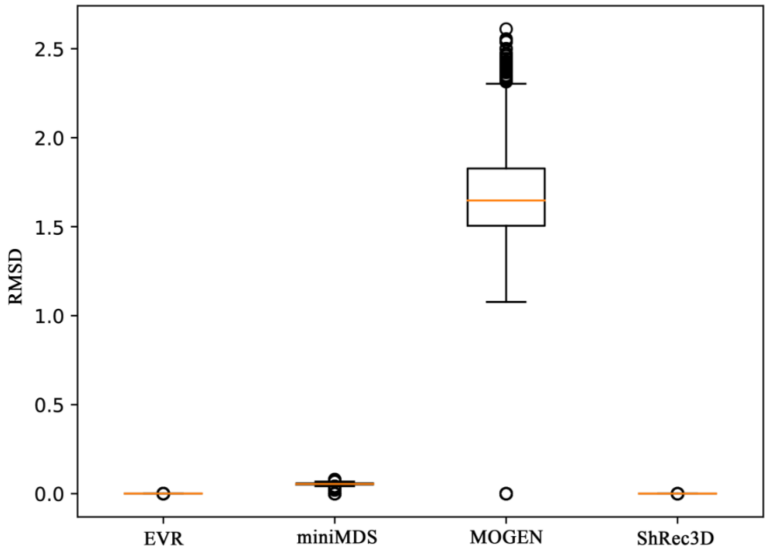

Figure S5. Evaluation for the dependency of the final structure on the randomly assigned initial conformation. The distribution of the RMSD values calculated based on pairwise comparisons of the final structures indicates the similarity between the final structures obtained from different initial (random) conformations. It can be seen that for ShRec3D and our EVR algorithm the final structures are highly similar to each other and thus not sensitive the random initial conformations.

To test if the final structures are sensitive to the randomly assigned initial conformations, the reconstruction processes of the standard toroidal spiral structure were repeated 100 times and the 100 final structures were compared in a pairwise way by calculating RMSD between every pair (classification of structures by mirror symmetry are considered if it is relevant). The distributions of the obtained RMSD values from the 4 software tools were plotted in **Figure S5**. As can be seen, the final structures are not sensitive to the initial random conformations in our EVR algorithm; so does ShRec3D, because no initial conformation is defined in its algorithm.

Fluorescent marker sites on the *E. coli* genome and their mutual distances

The data (**Table S1)** for the fluorescent marker sites on the *E. coli* chromosome and their experimentally measured mutual distances were compiled from published papers: Table S1 of (Espeli *et al.*, 2008) and Table S1 of (Lioy *et al.*, 2018). As shown in **Table S1**, the fluorescent marker names (Site name), marker positions on the linear genome (Site index), the mutual distance of marker site pairs (Distance) and the standard deviation in the measurement (SD) are listed. These fluorescent marker sites were mapped onto the 3D chromosome structure of *E. coli* reconstructed by EVR (**Figure S6**) and then the structure-based distances were calculated according to their 3D coordinates. The correlation between the structure-based distances and the experimentally measured distances was analyzed to show the accuracy of our EVR algorithm (see **Figure 5** in main text for more details).

Table S1. The experimentally measured distances of fluorescent marker sites on the *E. coli* chromosome

Site name	Site index	Site name	Site index	Distance (μm)	SD
left1	2616013	right2	738100	0.56	0.11
ori3	4413507	ter6	1689438	0.78	0.15
ori5	3909402	nsr2	258144	0.42	0.19
right1	602547	right2	738100	0.24	0.12
right2	738100	right5	1080438	0.28	0.13
right1	602547	right5	1080438	0.24	0.17
right2	738100	nsr1	71279	0.41	0.13
right2	738100	ori4	9883	0.42	0.18
right3	806549	right5	1080438	0.26	0.18
ter1	1308375	right5	1080438	0.41	0.28
ter1	1308375	ter9	1806680	0.17	0.08
ter2	1341067	right5	1080438	0.44	0.23
ter2	1341067	ter9	1806680	0.15	0.07
ter3	1395706	right2	738100	0.41	0.23
ter3	1395706	right4	1056444	0.34	0.20
ter3	1395706	ter6	1689438	0.08	0.04
ter3	1395706	ter1	1308375	0.12	0.06
ter3	1395706	ter2	1341067	0.10	0.04
ter3	1395706	ter3+10kb	1405706	0.04	0.02

ter3	1395706	ter9	1806680	0.16	0.07
ter4	1444252	ter1	1308375	0.13	0.10
ter4	1444252	ter2	1341067	0.12	0.10
ter5	1568683	left1close	2616013	0.51	0.12
ter5	1568683	left1far	2616013	0.61	0.09
ter5	1568683	ori4	9883	0.60	0.12
ter6	1689438	right4	1056444	0.36	0.16
ter6	1689438	right5	1080438	0.30	0.20
ter6	1689438	ter3	1395706	0.32	0.19
ter6	1689438	ter4	1444252	0.27	0.15
ter8	1891415	ter2	1341067	0.36	0.11
ter9	1806680	ter4	1444252	0.24	0.18
ter9	1806680	ter8	1891415	0.23	0.12

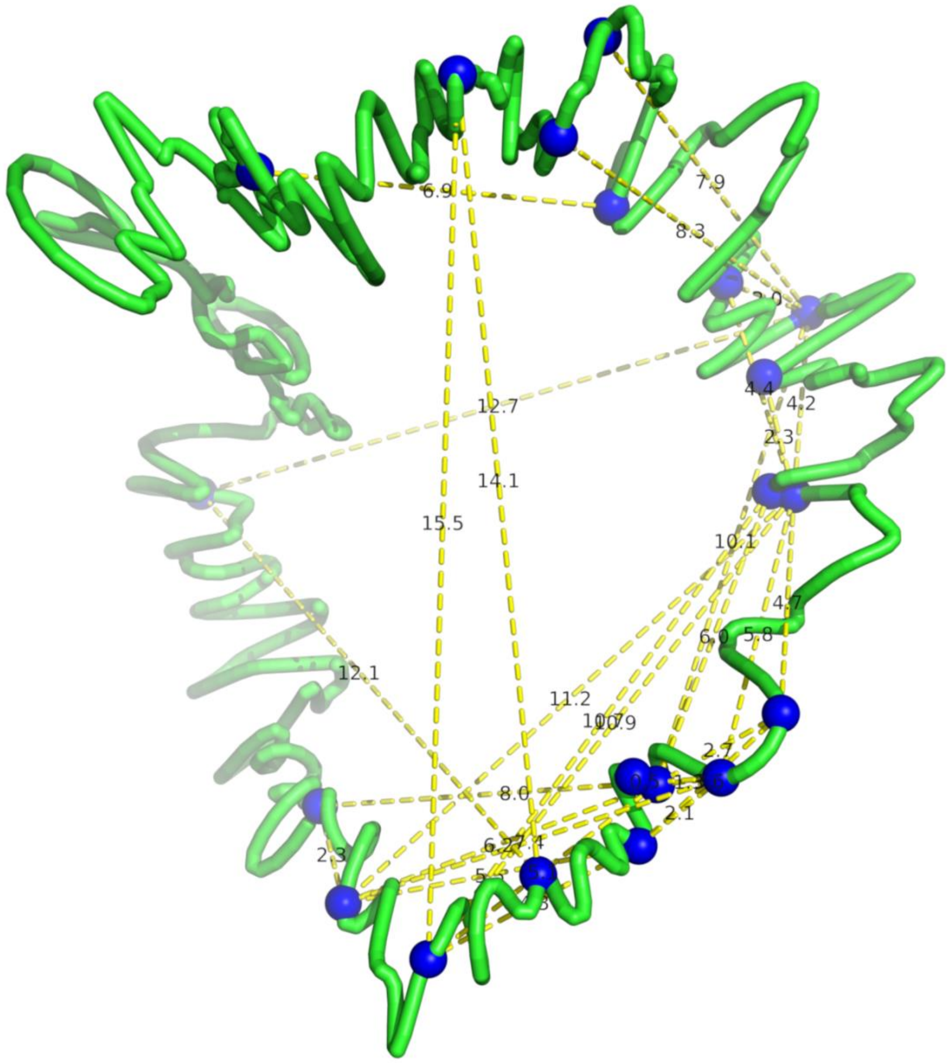

Figure S6. The distribution of fluorescent marker sites on the *E. coli* 3D chromosome structure reconstructed by EVR algorithm. The green tube is the chromosome structure; the blue beads are the fluorescent marker sites; the yellow dash lines show the mutual distances of fluorescent marker pairs.

Local optima analysis of our EVR algorithm

A general concern of optimization problem is falling into local optima. We checked this issue in our algorithm. As shown in **Figure S7**, local optima do exist in the optimization process of our algorithm. Nonetheless, it seems that our algorithm can break through the local optima and reach a global solution, possibly owing to its simple formulation.

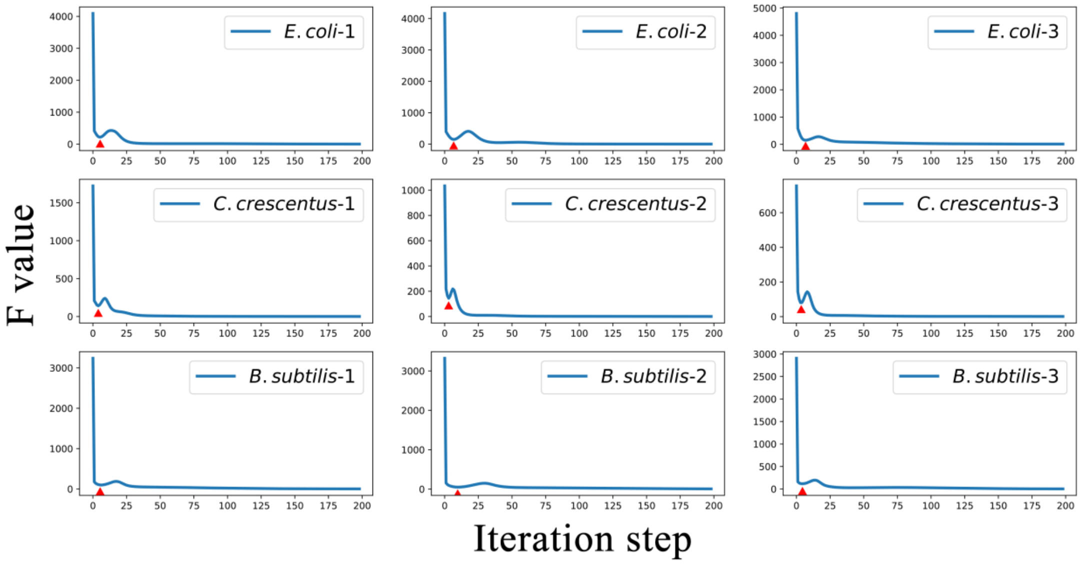

Figure S7. F value (see *Eq.* 5 in the main text) can break through local optimum to reach global minimum during optimization process. Red triangles indicate the local minima. The data used in the test are: *E. coli*-1 (GSM2870407), *E. coli*-2 (GSM2870414), *E. coli*-3 (GSM2870422), *C. crescentus*-1 (GSM1120445), *C. crescentus*-2 (GSM1120448), *C. crescentus*-3 (GSM1120450), *B. subtilis*-1 (GSM1671399), *B. subtilis*-2 (GSM1671405), *B. subtilis*-3 (GSM1671426), respectively.

References

Espeli,O. *et al.* (2008) DNA dynamics vary according to macrodomain topography in the *E. coli* chromosome. *Mol. Microbiol.*, **68**, 1418–1427.

Lioy,V.S. *et al.* (2018) Multiscale Structuring of the E. coli Chromosome by Nucleoid-Associated and Condensin Proteins. *Cell*, **172**, 771–783.e18.
